# A newborn with grouped facial skin lesions and subsequent seizures

**DOI:** 10.1186/1471-2431-14-126

**Published:** 2014-05-22

**Authors:** Michaela Döring, Karin Melanie Rohrer, Ilias Tsiflikas, Wolfgang Buchenau, Marko Wilke, Rupert Handgretinger, Christian Friedrich Poets, Rangmar Goelz

**Affiliations:** 1 Department I – General Paediatrics, Hematology/Oncology, University Hospital Tuebingen, Children’s Hospital, Hoppe-Seyler-Str.1, Tuebingen 72076, Germany; 2 Department of Radiology, Diagnostic and Interventional Radiology, University Hospital Tuebingen, Hoppe-Seyler-Str. 3, Tuebingen 72076, Germany; 3 Department IV – Neonatology, University Hospital Tuebingen, Children’s Hospital, Calwerstr. 7, Tuebingen 72076, Germany; 4 Department III – Neuropaediatrics, Developmental Neurology, Social Paediatrics, University Hospital Tuebingen, Children’s Hospital, Hoppe-Seyler-Str.1, Tuebingen 72076, Germany

**Keywords:** Congenital grouped skin lesions, Mixed connective tissue disease, Neonatal lupus erythematosus, Seizure

## Abstract

**Background:**

Congenital grouped skin lesions are alarming signs of a variety of threatening diagnoses of quite different origin. The present case report shows an impressive clinical pattern of a neonate and illustrates the difficulty in differential diagnosis of mixed connective tissue disease and neonatal lupus erythematosus in newborns. This reported case is to our knowledge the first description of an unrecognized mixed connective tissue disease in the mother with an unusual clinical manifestation in the newborn, comprising skin lesions, neurological damage and non-typical antibody constellation.

**Case presentation:**

We report on a Caucasian female neonate from a perinatally asymptomatic mother, who presented with grouped facial pustular-like skin lesions, followed by focal clonic seizures caused by multiple ischemic brain lesions. Herpes simplex virus infection was excluded and both the mother and her infant had the antibody pattern of systemic lupus erythematosus and neonatal lupus erythematosus, respectively. However, clinical signs in the mother showed overlapping features of mixed connective tissue disease.

**Conclusion:**

This case report emphasizes congenital Lupus erythematosus and mixed connective tissue disease as important differential diagnoses of grouped skin lesions in addition to Herpes simplex virus-infection. The coexistence of different criteria for mixed connective tissue disease makes it difficult to allocate precisely maternal and congenital infantile disease.

## Background

Congenital grouped skin lesions are alarming signs of a variety of threatening diagnoses of quite different origin [1]. The present case report shows the surprising resolution of an impressive clinical pattern of a neonate and his prenatally asymptomatic mother. Infectious diseases and typical neonatal patterns as well as auto-immunological entities have to be considered; especially in the latter, diagnosis may be difficult due to overlapping definitions.

## Case presentation

**Figure 1:**
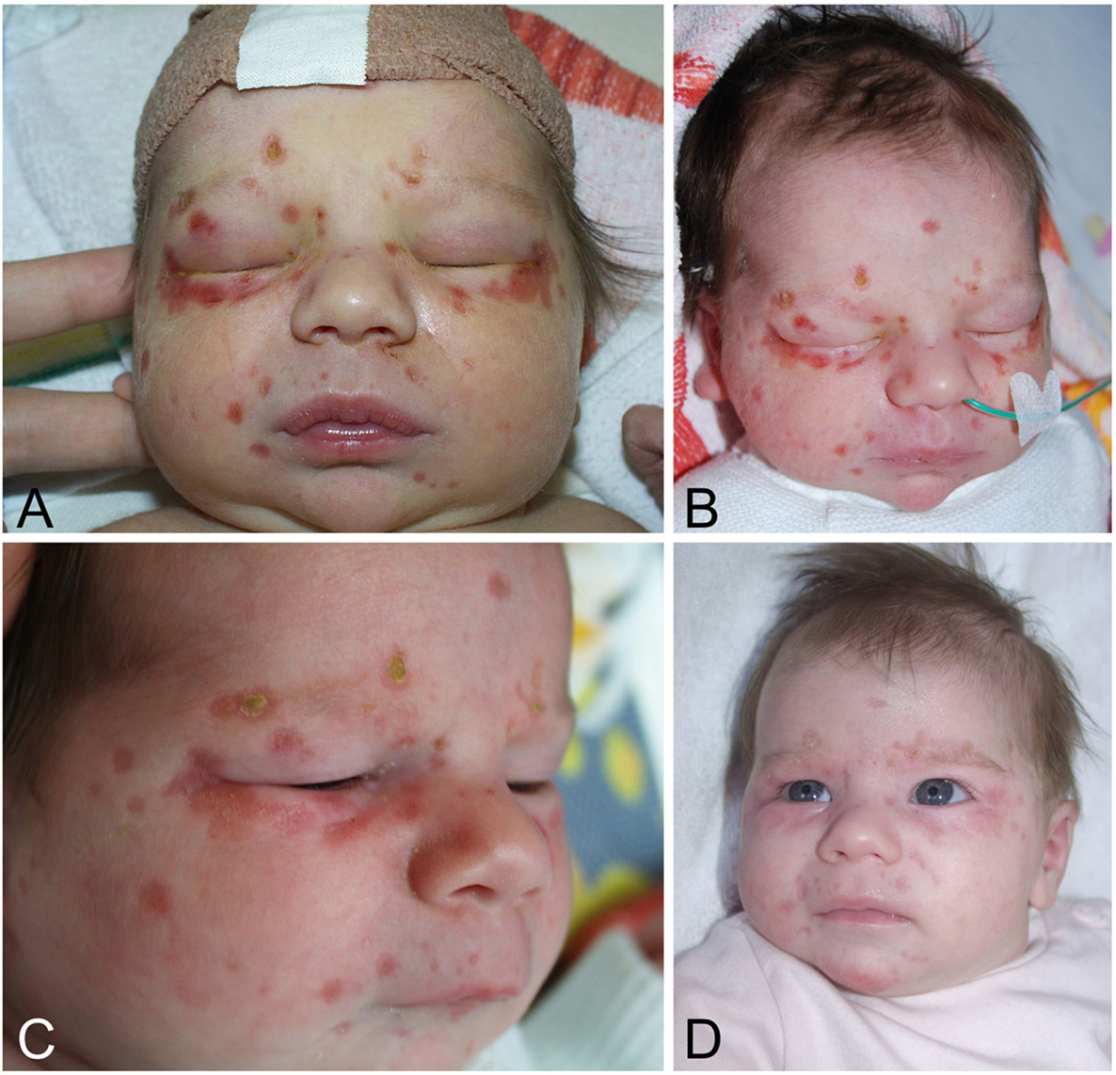
**Skin lesions and annular erythema.** Pustular lesions and annular erythema, partly with central lightening, yellowish, crusted plaques or slight scaling on the skin, exclusively in the face and on the forehead on the second day **(A and B)**, after one month **(C)**, and after three months **(D)**.

**Figure 2:**
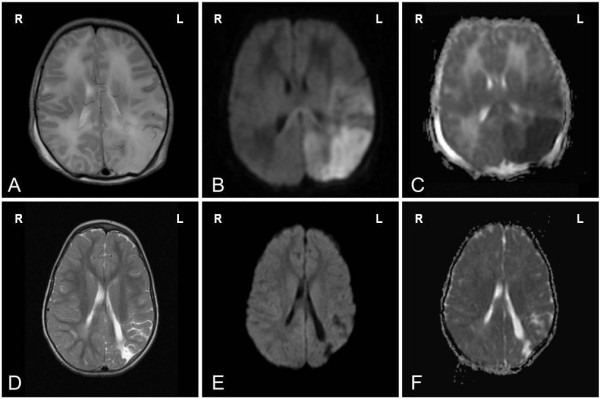
**MRI of ischemic lesions.** Abnormal signal changes in multiple brain areas due to ischemic lesions in the distribution of the middle and posterior cerebral artery on the left side. **A-C**: MRI on the 4^th^ postnatal day. **A**: High-signal intensity in T2w imaging corresponding to **B**: high-signal intensity in diffusion weighted imaging (DWI) (b1000) and **C**: low-signal intensity in apparent diffusion coefficient maps (ADC-MAP). **D-F**: MRI at 32 months. **D**: T2w imaging **E**: DWI.(b1000) and **F**: ADC-MAP showing now a scar formation on the left occipital lobe.

A term female infant was delivered by a 39 year old woman in a peripheral hospital after an uncomplicated 3^rd^ pregnancy with 41 week of gestational age. Delivery was assisted by vacuum extraction. Apgar score was 8/9/10 after 1/5/10 minutes respectively, and the infant had a birth weight of 2920 g (8^th^ percentile), a length of 50 cm (20^th^ percentile), and a head circumference of 35 cm (50^th^ percentile). The postnatal physical examination showed grouped pustular lesions and annular erythema, partly with central lightening, yellowish crusted plaques or slight scaling on the skin, exclusively in the face and on the forehead (Figure 1). The rest of the integument and the mucosa were inconspicuous. The child was transferred to a neonatal tertiary care center under the tentative diagnosis of a herpes simplex virus (HSV) infection, and antiviral therapy with aciclovir was promptly initiated. About six hours postnatal, the baby developed focal clonic seizures of the right arm. An electroencephalogram showed focal temporo-parieto-occipital changes over the left hemisphere with low amplitude, slight increase of slow waves and a reduced basic activity. Further, intermittent short-term focal rhythmic parietal theta activity on the left as well as rhythmic occipital delta activity on the left could be detected while no typical epileptic discharges occured. A lumbar puncture showed no sign of infection, with a normal protein and white blood count. In particular, HSV-PCR was negative. Anticonvulsive therapy with phenobarbital was initiated but seizures could only be controlled after adding phenytoin. A diffusion- weighted magnetic resonance imaging was performed on the 3^rd^ day of life, revealing multiple ischemic brain areas in the distribution of the middle and posterior cerebral artery on the left side (Figure 2A-C).

No evidence of sinus venous thrombosis was found. Thrombocytopenia (minimal 91000/μl) was present, and coagulation blood test showed evidence of an increased fibrinolysis and normal plasmatic inhibitors of coagulation, normal antithrombin III (56%) and protein S (58%) but low protein C (22%). Therapy with low molecular weight heparin was started. Echocardiogram revealed no structural abnormalities, and an inconspicuous electrocardiogram with a normal PQ interval and QRS complex was analyzed. Central and peripheral bleeding spots were found in the ocular fundus, most likely caused by trauma during birth. Renal function test, albumin, transaminases, bilirubin and calcium were normal. A smear of the grouped pustular lesions in the face showed no infection with staphylococcus. But a biopsy of the affected skin showed evidence of cytoid bodies compatible with an interface dermatitis. Serological studies of the infant detected maternal HSV and varicella zoster virus (VZV) specific IgG antibodies, IgM and a HSV-PCR of a swab of a skin lesion were negative. However, antinuclear antibodies (ANA), anti-U1-snRNP antibodies and anti-Sm antibodies were present, while antiphospholipid, anti-Scl 70, SSA/Ro, SSB/La, anti-Tm, anti-Jo-1 and anti-ds DNA antibodies could not be detected. Test results of the asymptomatic mother revealed the same autoantibody pattern.

Four weeks postpartum, the mother developed bilateral erythema on the face and both forearms, muscle weakness of both arms, a Raynaud’s phenomenon, arthralgia in hands and swollen fingers. She was subsequently diagnosed with mixed connective tissue disease (MCTD), since she exhibited three typical clinical symptoms (swollen fingers, muscle weakness, Raynaud’s phenomenon), and one of the possible antibody constellations (ANA, anti-U1-snRNP and anti-Sm antibodies), both characteristic signs and symptoms of the disease [2,3]. There had been no occurrence of symptoms of rheumatic diseases in the infant’s siblings, a seventeen years old sister and a seven years old brother. Further questioning of the mother revealed that about six weeks after the second delivery, transient arthralgia occurred, which responded to oral steroids. No diagnosis was made at that time.

The newborn infant was discharged on his 16^th^ postnatal day, the lesions were healing (in parts showing scars as residual lesions), and until reassessment at the age of 10 weeks with phenobarbital and low molecular weight heparin as medical therapy displayed no symptoms; he was consequently carefully weaned off medication. A transcranial duplex ultrasound after 4 weeks of birth showed a normal cerebral artery blood flow in all areas of the brain. A follow-up MRI of the brain (Figure 2D-F) at the age of 32 months showed postischemic defects with involvement of the gyrus supramarginalis and the peripheral visual cortex. The rest of the brain showed age-appropriate myelinization and no further over lesions. At the age of 6 years there were no signs of cerebral palsy, seizures or amblyopia but language development was delayed and complicated by a speech disorder. Other cognitive development as tested by the Snijders-Oomen non-verbal intelligence test was in the lower norm and a somatic retardation with dystrophy and microcephaly was diagnosed.

In summary, we describe a newborn infant who had cutaneous manifestations and the antibody pattern of neonatal lupus erythematosus (NLE), complicated by neurological symptoms.

## Conclusions

This case report emphasizes NLE/MCTD as an important differential diagnosis of grouped skin lesions in addition to HSV-infection, which may ultimately be a sign of several conditions (see Table 1 for an overview). However, the coexistence of several sets of classification criteria for MCTD makes it difficult to allocate precisely maternal and congenital infantile disease.

**Table 1:** Differential diagnosis of immune mediated disorders presenting with neurological symptoms and skin lesions

	**Clinical characteristics in newborn patients with skin and neurological lesions**		
**Diagnosis**	**Skin**	**Neurological characteristics**	**Serological characteristics**
**Neonatal Herpes Simplex Virus 1 (HSV1)**	vesiculopustular lesions	seizures, tremors, lethargy	HSV1-IgM antibodies positive
**Congenital Varicella Syndrome (VZV)**	skin rashes, papules, vesiculopustular lesions, crusts	seizures	VZV-IgM antibodies positive
**Herpes Zoster (HZ)**	skin rash, vesicles, crusts, limited to a dermatome	seizures	VZV-IgM antibodies positive
**Neonatal Lupus Erythematosus (NLE)**	malar rash or butterfly rash, scaly patches, skin and mucosa lesion like ulcers	seizures	ANA, anti- Sm antibodies, SSA/Ro and SSB/La antibodies, anti-U1-RNP antibodies
**Mixed Connective Tissue Disease (MCTD)**	rash, scaly patches, overlapping symptoms of SLE, PSS, PM and RA	-	ANAs, anti-U1-RNP antibodies, (anti-Sm antibodies)
**Undifferentiated Connective Tissue Disease (UCTD)**	malar rash, oral ulcers purpura, urticaria	seizures, neuropathy	ANA, anti-Ro/SSA, anti-Sm-, anti-ds DNA-, anti RNP-, anti-Ku antibodies, rheumatoid factor
**Antiphospholipid Syndrome (APS)**	skin ulcers	seizures, strokes, abnormal movements	antiphospholipid antibodies, β2- glycoprotein- 1 antibodies, anticardiolipin antibodies
**Incontinentia Pigmenti (IP)**	blistering, a wart-like rash, hyper/hypopigmentation	cerebral atrophy, seizures, slow motor development, muscle weakness	NEMO IKBKG gene (chromosomal locus Xq28), females skewed X-Chromosome inactivation
**Erythema Multiforme (EM)**	pink-red blotches, target lesions	seizures (caused by HSV)	viral, bacterial or fungal infection
**Neonatal Acne (NA)**	comedones, papules, pustules	-	*Malassezia* species
**Langerhans Cell Histiocytosis (LCH)**	scaly erythematosus lesions, red papules, eruptions on the scalp	-	
**Impetigo Contagiosa (IC)**	honey yellow crusts, plaques or bullae	-	*Staphylococcus aureus*, *beta-hemolysing Streptococcus, group A*
**Congenital Syphilis(CS)**	pemphigus syphiliticus	seizures, pseudoparalysis	*Treponema pallidum*

Based on the initial clinical presentation, the first differential diagnosis focused first on neonatal HSV infection and, less probable, congenital VZV infection. Thus, aciclovir therapy (3x20 mg/kg/d) was started immediately. Seizures just six hours after birth further supported this hypothesis. HSV infection in neonates is rather uncommon and might be difficult to diagnose [4]. Vesicular eruptions and seizures can occur at any time from soon after birth up to beyond the neonatal period [5]. Congenital infection is usually identified within the first 48 hours following birth, characterized by skin vesicles or scarring, eye lesions, neurologic symptoms, and later growth retardation and psychomotor developmental retardation [6]. Therefore, therapy in the present case had to be continued until acute HSV and VZV infection had been excluded [7,8] by serologic and PCR investigations. At his point, the less-common NLE, MCTD, erythema multiforme (EM), antiphospholipid syndrome (APS) and incontinentia pigmenti (IP) were considered. In general, vesiculopustular eruptions in neonates are common in bacterial infections like staphylococcal infections, congenital lues or listeriosis, fungal infections (candidiasis) or parasitic disorders (scabies) [1]. Transient pustular melanosis is characterized by eosinophilic staining. EM is a short-lasting skin condition that occurs as a reaction to a viral infection (especially HSV) or medication. In the present case, these diagnoses could be excluded clinically, due to the normal microbial flora of skin, and by a negative C-reactive protein, which is highly sensitive to bacterial infection [9]. The analyzed skin biopsy made EM and IP unlikely [10-12]. In contrast to these more common causes, immune-mediated disorders manifesting with neurological symptoms (such as seizures) in newborns are extremely rare [13,14]. Several cases of NLE-associated seizures are reported [15-20]. NLE is an acquired autoimmune disease caused by maternal antibodies to SSA /Ro and SSB/La, ANAs, anti-ds DNA or anti-Sm antibodies and in rare cases anti-RNP antibodies [21]. Maternal anti-Ro/SSA and anti-La/SSB antibodies are known to be associated with NLE including fetal cardiac conduction block [22]. All infants who were affected with NLE and tested positive only for anti-U1-snRNP antibodies were male, only developed cutaneous lesions and were not commonly associated with congenital heart block [13,23]. In about 99% of SLE-patients, antinuclear antibodies were detected [24,25]. In most cases, the presence of anti-Sm antibodies occurs after the clinical manifestation of SLE therefore they serve as a diagnostic criterion for SLE, while a high anti-U1-snRNP antibody titer is associated with a more active illness. Nevertheless, mild progressive forms often occur without nephritis [3]. Ultrasound scans showed normal structure and perfusion of both kidneys in the present case.

The cause of the multiple ischemic brain areas in the distribution of the middle and posterior cerebral artery on the left side remains unclear. The homeostasis system of neonates is characterized by decreased concentration of protein S, protein C and antithrombin, elevated levels of factor VIII and von Willebrand factor, and less active fibrinolysis, which results in a prothrombotic state. Neonatal thrombosis is a multifactorial event including several inherited and acquired risk factors such as hereditary deficiencies of the naturally occurring coagulation inhibitors, maternal preeclampsia, traumatic delivery, infection, dehydration, hyperviscosity, complex heart disease and catheter placement in the newborn [26,27]. The activation of protein C plays a central role in the prophylaxis of micro-thrombi. Activated protein C is able to form complexes with its cofactor protein S which may then inactivate the coagulation factors Va and VIIIa [28]. The importance of the function of protein C is shown by the life threatening thrombotic complications in newborn infants. In the process, there are two different forms of protein C deficiencies: type I is accompanied by reduced synthesis of protein C thus reduced plasma levels, while type II is characterized by reduced protein C activity but normal plasma level. Newborns born with a proven homozygous type I deficiency experience severe thrombosis and purpura fulminans shortly after birth. The prevalence of heterozygous protein C-deficiency (activity 30-65%) ranges from 1:200 to 1:500 [29-31]. In the present case, the female infant had a mild deficiency of protein C (22%) [32,33]. Ultimately, a combination of an autoimmune phenomenon and a heterozygous protein C deficiency likely leads to the unusual neurologic manifestations in our patient.

The clinical presentation of the patient’s mother four weeks postpartum was typical for MCTD but her antibody constellation was typical for SLE [34]. MCTD was originally described by Sharp et al. in 1972 and is classically considered as an overlap syndrome presenting with features of SLE, progressive systemic sclerosis, polymyositis, and rheumatoid arthritis. Anti-U1-RNP antibodies are detectable in the blood of all MCTD patients, typically showing high titres of ANAs but without additional antibodies like DNA antibodies of SLE and the Scl-70 antibodies typical for scleroderma [3,35]. Giving a precise definition of MCTD is difficult since there are four different sets of criteria which are generally used in international publications: the Sharp criteria, the Alarcon-Segovia criteria, the Kasukawa criteria, and the Kahn criteria [36]. The basis of all these sets of criteria is the presence of anti-U1-RNP autoantibodies. Comparing the four sets of classification criteria, Amigues et al. concluded that the criteria with the best performance are those proposed by Alarcon-Segovia [35]. These criteria are widely used and do not consider the positivity to Sm antigen as an exclusion criterion like the Sharp criteria do [3,37]. They include five clinical symptoms (swollen hands, synovitis, biologically proven myositis, Raynaud’s phenomenon, and acrosclerosis with or without proximal systemic sclerosis) in addition to anti-RNP positivity. At least three out of five clinical symptoms and the presence of anti-RNP antibodies are necessary for diagnosis of MCTD [2,35]. This allows the diagnosis of MCTD in the mother of the patient.

In conclusion, the diagnosis in the present case was difficult not only due to the exceedingly rare presentation of such diseases in the neonatal period but also because the mother’s autoimmune disease had not been diagnosed at the time of delivery. This reported case is to our knowledge the first description of an unrecognized MCTD in the mother with an unusual clinical manifestation in the newborn, comprising skin lesions, neurological damage and non-typical antibody constellation. It illustrates the diagnostic difficulties in a symptomatic child born to an asymptomatic mother, and underlines that the neonatal manifestations of an autoimmune disease may differ dramatically from those observable in the mother.

## Consent

Written informed consent was obtained from the patient’s parents for publication of this Case Report and any accompanying images. A copy of the written consent is available for review by the Editor-in-Chief of this journal. The present case report was performed in accordance with the institutional ethics regulations.

## Abbreviations

ANA: Antinuclear antibodies; APS: Antiphospholipid syndrome; EM: Erythema multiforme; HSV: Herpes simplex virus; IP: Incontinentia pigmenti; MCTD: Mixed connective tissue disease; NLE: Neonatal lupus erythematosus; SLE: Systemic lupus erythematosus; VZV: Varicella zoster virus.

## Competing interests

The authors declare that they have no competing interests.

## Authors’ contributions

All authors have participated in case report design, interpretation, and writing of the report. MD collected the data of the case report, review of literature, and drafted the first version of the manuscript. KMR, IT and WB collected the data of the case report and wrote a part of the manuscript. MW, RH and CFP reviewed the manuscript. RG primarily participated in case report design and helped to draft the manuscript. All authors read and approved the final manuscript.

## Pre-publication history

The pre-publication history for this paper can be accessed here:

http://www.biomedcentral.com/1471-2431/14/126/prepub
